# Local and Systemic Changes in Photosynthetic Parameters and Antioxidant Activity in Cucumber Challenged with *Pseudomonas syringae* pv *lachrymans*

**DOI:** 10.3390/ijms21176378

**Published:** 2020-09-02

**Authors:** Tomasz Kopczewski, Elżbieta Kuźniak, Andrzej Kornaś, Grzegorz Rut, Michał Nosek, Iwona Ciereszko, Lech Szczepaniak

**Affiliations:** 1 Department of Plant Physiology and Biochemistry, Faculty of Biology and Environmental Protection, University of Łódź, Banacha 12/16, 90-237 Łódź, Poland; tomekop@gmail.com; 2 Institute of Biology, Pedagogical University, Podchorążych 2, 30-084 Kraków, Poland; andrzej.kornas@up.krakow.pl (A.K.); grzegorz.rut@up.krakow.pl (G.R.); michal.nosek@up.krakow.pl (M.N.); 3 Department of Plant Biology and Ecology, Faculty of Biology, University of Bialystok, Ciolkowskiego 1J, 15-245 Bialystok, Poland; icier@uwb.edu.pl; 4 Department of Environmental Chemistry, Faculty of Chemistry, University of Bialystok, Ciolkowskiego 1K, 15-245 Bialystok, Poland; lech@uwb.edu.pl

**Keywords:** ascorbate–glutathione cycle, chloroplasts, gas exchange, photochemical activity, redox signature, superoxide dismutase, tocopherols

## Abstract

We studied changes in gas exchange, photochemical activity and the antioxidant system in cucumber leaves locally infected with *Pseudomonas syringae* pv *lachrymans* and in uninfected systemic ones. Infection-induced declined net photosynthesis rate and the related changes in transpiration rate, the intracellular CO_2_ concentration, and prolonged reduction in maximal PSII quantum yield (Fv/Fm), accompanied by an increase in non-photochemical quenching (NPQ), were observed only in the infected leaves, along with full disease symptom development. Infection severely affected the ROS/redox homeostasis at the cellular level and in chloroplasts. Superoxide dismutase, ascorbate, and tocopherol were preferentially induced at the early stage of pathogenesis, whereas catalase, glutathione, and the ascorbate–glutathione cycle enzymes were activated later. Systemic leaves retained their net photosynthesis rate and the changes in the antioxidant system were partly like those in the infected leaves, although they occurred later and were less intense. Re-balancing of ascorbate and glutathione in systemic leaves generated a specific redox signature in chloroplasts. We suggest that it could be a regulatory element playing a role in integrating photosynthesis and redox regulation of stress, aimed at increasing the defense capacity and maintaining the growth of the infected plant.

## 1. Introduction

Reactive oxygen species (ROS) and redox signaling play multiple roles in plant biology by mediating metabolic, growth and developmental processes as well as plant response to stress [1,2,3]. Most environmental stressors, including pathogens, disturb the cellular redox environment which is buffered by the main soluble redox couples, i.e., reduced and oxidized ascorbate (AA/DHA), reduced and oxidized glutathione (GSH/GSSG) as well as reduced and oxidized nicotinamide adenine dinucleotide phosphate (NADPH/NADP). The redox homeostasis is largely influenced by ROS, including those generated during photosynthesis and other metabolic processes, which are overproduced under stress conditions. The amplified ROS production is counterbalanced by the ROS-processing system which determines the localization as well as the extent and specificity of ROS/redox signals, known as the ROS/redox signature [4].

Rapid generation of ROS, mainly superoxide anion radical (O_2_^−^) and H_2_O_2_, is one of the earliest plant responses to pathogens observed in numerous plant–pathogen interactions [5,6,7]. Among ROS, H_2_O_2_ which is a relatively stable and non-charged molecule, is considered a versatile signaling molecule involved in the intracellular communication system that activates stress responses, including redox-dependent reprogramming of defense gene expression [8,9]. 

The ROS-processing systems such as the antioxidant enzymes, the ascorbate–glutathione cycle and tocopherols, have been shown to play an important role in managing ROS, particularly H_2_O_2_, generated at the sites of infections and in initiating signals eliciting downstream defense reactions at the local and systemic levels [10,11]. The ascorbate–glutathione cycle is an antioxidant system, but it also fulfils regulatory functions in redox signaling. The three major redox metabolites in plant cells, ascorbate, glutathione, and pyridine nucleotides are linked through the ascorbate–glutathione cycle which regulates their redox signaling potential simultaneously determining the ROS signature [2]. The changed activity of the ascorbate–glutathione cycle was reported in plants subjected to abiotic stresses and in defense against pathogens [6,12,13]. In the tomato–*Botrytis cinerea* pathosystem, the development of disease symptoms was accompanied by a significant shift of the cellular redox balance towards the oxidative state and correlated with the decrease in concentrations and redox ratios of ascorbate and glutathione as well as insufficient activity of the ascorbate–glutathione cycle enzymes [14]. Similar changes were induced by *Pseudomonas syringae* infection in *Arabidopsis thaliana* plants [15].

The specificity of ROS/redox signal-induced plant stress response is compartment-dependent [16,17]. Chloroplasts which are organelles strictly specialized for conducting photosynthetic processes, also play a fundamental role in plant immunity. Pathogen recognition at the plasma membrane is signaled to chloroplasts within minutes, and H_2_O_2_ from the oxidative burst in the apoplast, Ca^2+^ as well as mitogen-activated protein kinase (MAPK) are likely candidates which transduce the biotic stress signal to chloroplasts [18]. Inhibition of photosynthetic activity, shown by chlorophyll fluorescence measurements and proteomic analysis, is a well-known phenomenon in plants infected by pathogens [19,20]. It has been recognized as an active plant defense mechanism contributing to the basal pathogen-associated molecular patterns (PAMP)-triggered immunity (PTI) as well as effector-triggered immunity (ETI), and light-induced ROS accumulation in chloroplasts is essential to hypersensitive response (HR) and defense gene expression [21,22].

Plant defense signaling and the outcome of plant–pathogen interaction are influenced by the chloroplast-to-nucleus retrograde communication coordinating the whole-cell responses. Chloroplast-derived H_2_O_2_ has been suggested to be a retrograde signaling molecule directly transferred to the nucleus via stromules [23]. Moreover, chloroplasts are involved in the biosynthesis of salicylic (SA), abscisic and jasmonic acids, stress hormones tightly linked with defense against pathogens at the whole plant level [24]. Finally, considering their internal redox environment, chloroplasts significantly contribute to cellular redox status and generate multiple ROS/redox defensive signals regulating the intracellular and intercellular immune responses [25,26,27,28]. For example, changes in cellular redox status control the conformation of NPR1 (NON-EXPRESSOR OF PATHOGENESIS-RELATED GENE 1), a master protein regulator of SA-dependent defense genes which are relevant to both local defense against biotrophic/hemibiotrophic pathogens and systemic acquired resistance [29]. Moreover, modulation of chloroplastic ROS production and the redox status of chloroplasts turned out to be a strategy of invading pathogens to increase their infection success [30]. Coronatine, a phytotoxin promoting *P. syringae* virulence in plants, may function to reduce photosynthetic efficiency and increase chloroplast localized ROS [20]. In *Puccinia striiformis* f. sp. *tritici*–wheat interaction, a fungal effector secreted from haustoria is translocated into plant chloroplasts, decreases photosynthetic electron transport rate and H_2_O_2_ generation in these organelles which inhibit plant defense responses at the sites of infection [31].

During biotic stress, the metabolic state of local (i.e., infected) and systemic plant parts is severely affected. Metabolic responses induced by a pathogen at the local leaf could be transmitted throughout the plant and provoke re-organization of the metabolism which induces a primed state in the systemic leaves [32]. The ROS-antioxidants interaction is considered an interface between stress sensing and the metabolic responses which influences plant defense processes [33]. Moreover, plant cells are envisaged to use ROS-induced ROS release for cell-to-cell communication transmitting the local stress signal throughout the plant via the auto-propagating ROS waves [34] Considering the importance of ROS/redox regulations in gene expression, control of metabolism and defense signaling, the changes in cellular redox state could be instrumental for our understanding of local and systemic plant defense responses [35]. 

*P. syringae* pv *lachrymans* (*Psl*) is a hemibiotrophic bacterial pathogen causing angular leaf spot on cucurbits, but it is most common on cucumber [36]. Using cucumber—*Psl* pathosystem, we studied how the photochemical activity of photosynthesis and the antioxidant/redox status in the yet uninfected systemic cucumber leaves changed after local *Psl* infection, and how these changes differ from those induced locally in the infected leaves. To gain insight into the role of chloroplasts in plant–pathogen interactions, the antioxidant/redox changes were determined at the whole cell and the chloroplast levels.

## 2. Results

**Figure 1:**
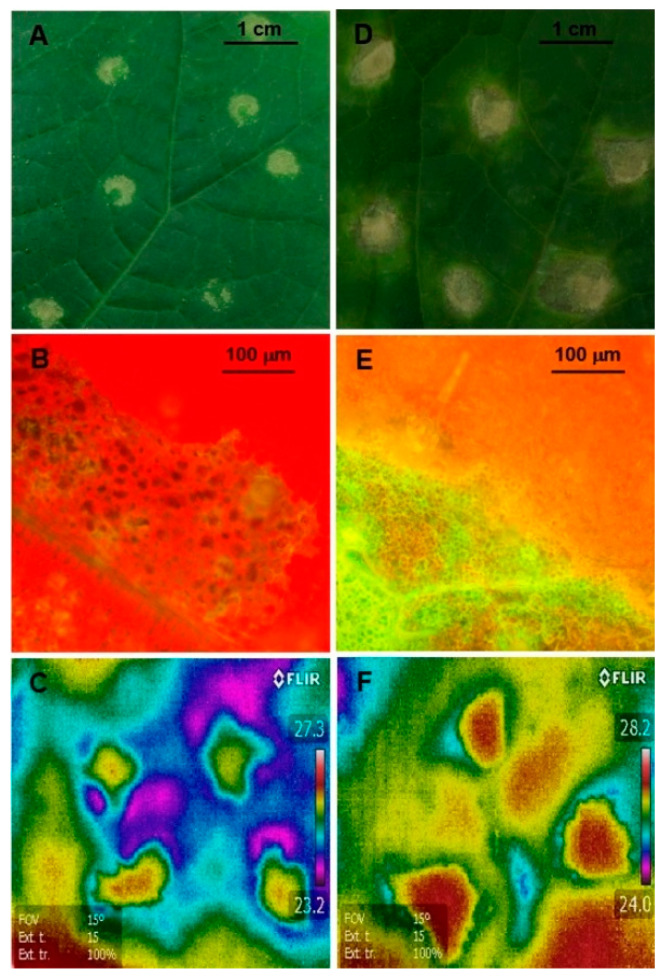
*Pseudomonas syringae* pv *lachrymans* (*Psl*) infection development in the 3rd leaves. (**A**,**D**) Macroscopic symptoms of *Psl* infection 2 dai after inoculation (**A**) and 7 dai (**D**). (**B**,**E**) Chlorophyll autofluorescence 2 dai (**B**) and 7 dai (**E**). (**C**,**F**) Thermal imaging of infection development 2 dai (**C**) and 7 dai (**F**). Images are representative for at least three experiments, using a minimum of six plants.

The angular leaf spot disease symptoms appeared in the inoculated leaves at 1–2 dai (days after inoculation) after inoculation (dai) and consisted of chlorotic spots at the sites of inoculation (Figure 1A). During the following 5 dai, these symptoms turned into angular necrotic spots surrounded by extensive chlorotic halo (Figure 1D). In the control, small yellowish spots were visible at the sites of water infiltration (data not shown). The infection was restricted to the *Psl*-inoculated 3rd leaf and it never entered the stem or other leaves. The development of necrotic lesions in the 3rd leaf was accompanied by significant decrease in chlorophyll autofluorescence (Figure 1B,E). This coincided with changes in the leaf surface temperature. Thermal imaging revealed the heterogeneity in infection-induced changes in leaf transpiration. The *Psl* inoculation sites in the 3rd leaf first became apparent as regions of lower temperature (Figure 1C). The extension of the cooling effect decreased as the necrotic lesions started to dry and expanding hot-spots were visible at 7 dai (Figure 1F).

### 2.1. Chlorophyll Fluorescence

**Figure 2:**
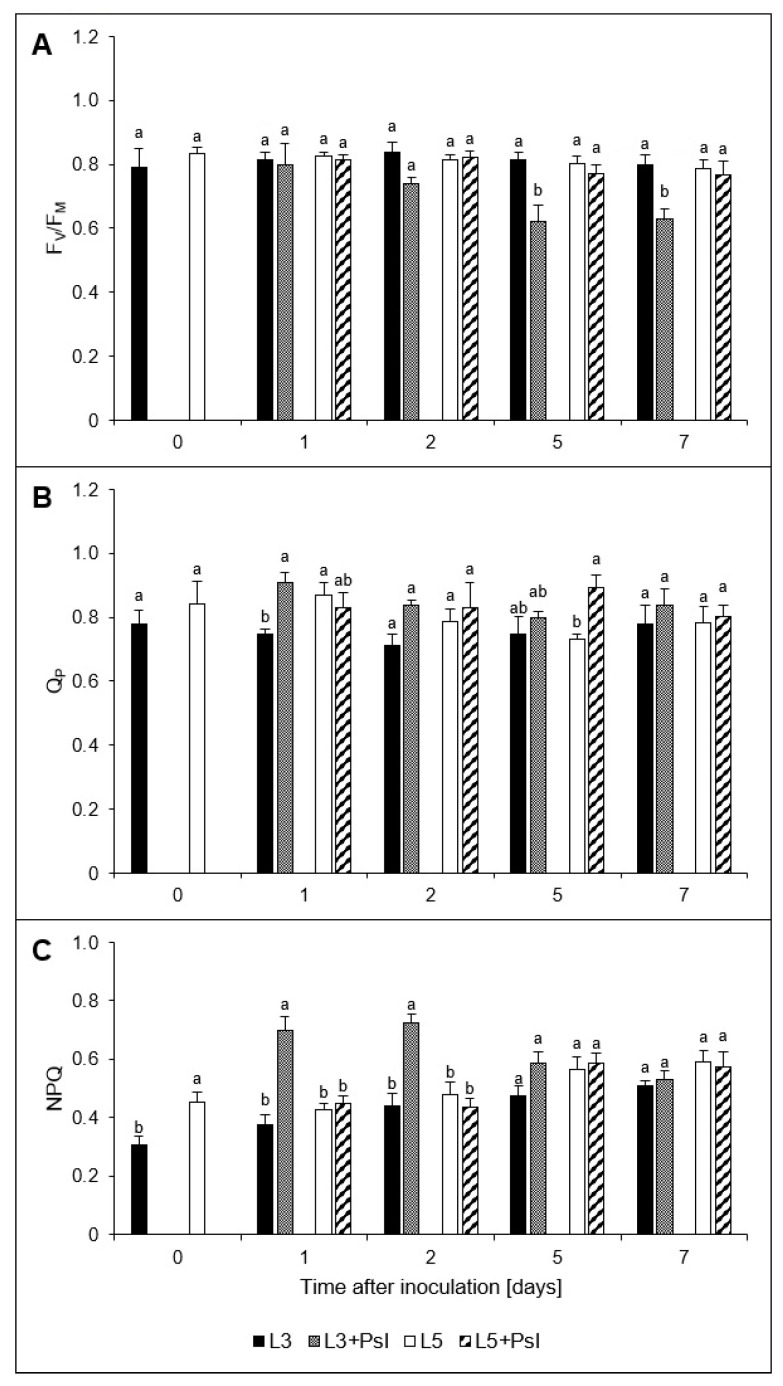
Chlorophyll fluorescence parameters: (**A**) the maximal yield of PSII (*F_v_*/*F_m_*), (**B**) the coefficient of photochemical quenching (*Q*_P_), (**C**) non-photochemical quenching (NPQ). Values are means of four replicates (±SD). Different letters (a,b) indicate significant (*p* ≤ 0.05) differences between experimental variants within a given time point.

**Figure A1:**
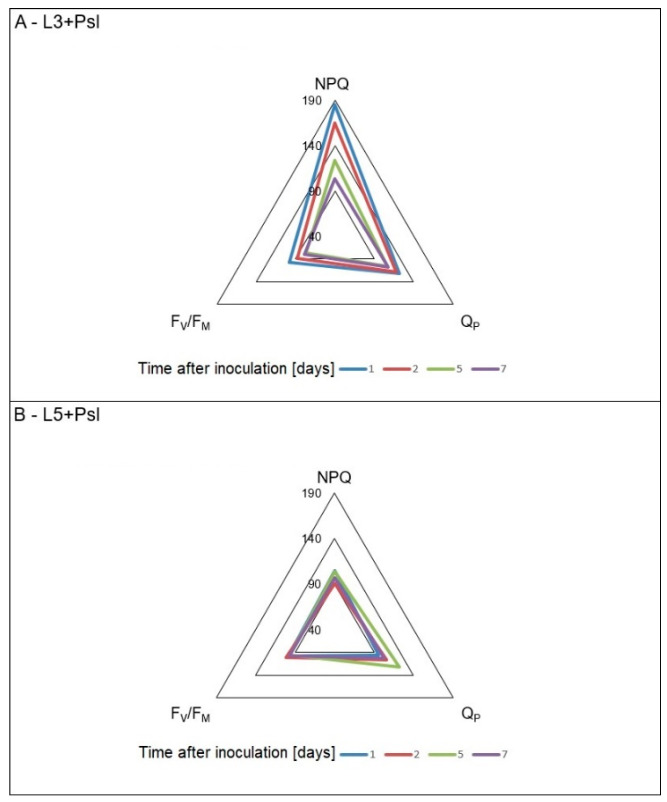
Relative changes of mean values of fluorescence parameters in cucumber plants infected with *Psl*. (**A**) The 3rd inoculated leaves; (**B**) the 5th non-inoculated leaves collected from the inoculated plants. All fluorescence data relative to respective control plants.

At the time point of inoculation, the 3rd and 5th leaves did not differ with respect to *F_v_*/*F_m_* and Q_P_ (Figure 2A,B). Infection induced a significant decrease in *F_v_*/*F_m_* in the 3rd leaves and at 7 dai, when fully developed disease symptoms were observed, *F_v_*/*F_m_* was 32% lower than in the control. In the 5th leaves of the infected plants, *F_v_*/*F_m_* values did not change when compared to the control and they were considerably higher than in the 3rd ones. NPQ processes were highly enhanced in the infected 3rd leaves, especially when the disease symptoms appeared in the leaves (2 dai) (Figure 2C). In the 5th leaves of the infected plants, NPQ did not change in relation to the control. However, at 2 dai its value remained significantly lower than in the 3rd ones. There were about 22% higher level of *Q*_P_ in the 3rd leaves after infection at 1 dai as well as in the 5th leaves at 5 dai compared to the adequate control. These pathogen-induced changes elicited distinct fluorescence signature characterizing the 3rd and 5th leaves of the inoculated plants (Figure A1).

### 2.2. Gas Exchange

**Figure 3:**
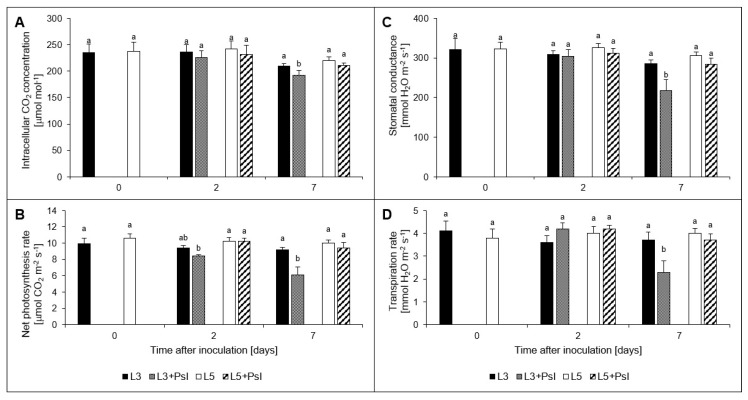
Gas exchange parameters: (**A**) intracellular CO_2_ concentration (C*_i_*), (**B**) net photosynthesis rate (*P_N_*), (**C**) stomatal conductance (G*_S_*), (**D**) transpiration rate (*E*). Values are means of four replicates (±SD). Different letters (a,b) indicate significant (*p* ≤ 0.05) differences between experimental variants within a given time point.

The patterns of infection-induced changes found for net photosynthesis rate, stomatal conductance and transpiration rate were similar, except a subtle increase in transpiration rate in the 3rd leaves after infection at 2 dai compared to the control. They decreased considerably in the infected 3rd leaves at 7 dai and did not change in the 5th ones (Figure 3B–D). The intracellular CO_2_ concentration was lower in the 3rd leaves after infection only at 7 dai (Figure 3A). 

### 2.3. SOD and CAT Activities 

**Figure 4:**
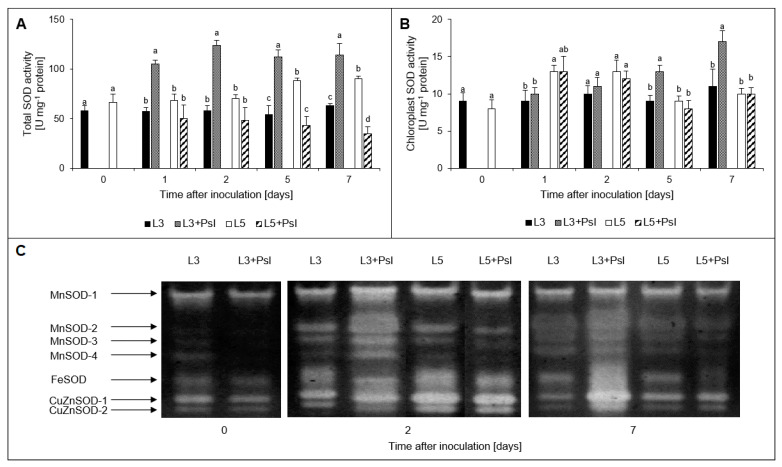
Superoxide dismutase (SOD) activity: (**A**) total SOD activity; (**B**) chloroplast SOD activity; (**C**) SOD isoforms electrophoregram. Values are means of four replicates (±SD). Different letters (a,b) indicate significant (*p* ≤ 0.05) differences between experimental variants within a given time point.

In the infected 3rd leaves, total SOD was substantially induced and an over 2-fold increase in the total SOD activity during the experimental period was found (Figure 4A). However, in the 5th leaves of the infected plants, the total SOD activity was found to decrease, down to 39% of the control on 7 dai. In the control plants, age-dependent SOD activity increase was observed in the 5th leaves and on the 5 and 7 dai it was 63% and 43% higher than in the 3rd leaves, respectively. The total SOD activity in chloroplasts isolated from the 3rd leaves tended to increase after infection whereas that in the 5th leaves remained unchanged. The strongest increase in the infected 3rd leaves was observed on the 5 and 7 dai, when total chloroplast SOD activity was 44% and 55% higher than in the non-infected plants, respectively. At these time points, in the infected plants, the levels of total SOD activity in chloroplasts of the 3rd leaves were significantly higher than those of the 5th leaves (Figure 4B). The in-gel activity assay with selective inhibitors (KCN and H_2_O_2_) allowed to identify four MnSOD, one chloroplast FeSOD, and two CuZnSOD isoforms (Figure 4C). In the 3rd leaves, the post-infectious increase in total SOD activity measured in the whole leaf extract at 2 dai was concomitant with the induction of the chloroplast-specific FeSOD and all MnSOD isoforms whereas at 7 dai, it was mainly due to the enhanced activities of CuZnSODs and FeSOD. In the 5th leaves, no significant infection-induced changes in SOD isoforms were observed, except for FeSOD activity which was visibly decreased in comparison to the control plants at 7 dai (Figure 4C and Table A1).

**Figure 5:**
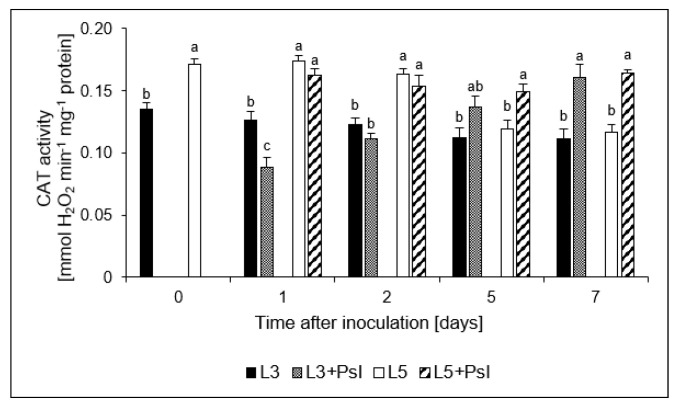
Catalase (CAT) activity. Values are means of four replicates (±SD). Different letters (a,b) indicate significant (*p* ≤ 0.05) differences between experimental variants within a given time point.

CAT activity showed biphasic changes in the infected 3rd leaves; the initial activity decrease at 1 dai was followed by an increase at the advanced stage of pathogenesis at 5 dai. In the 5th leaves, the infection-induced CAT activity increase was found only on the 5 dai (Figure 5).

### 2.4. The Ascorbate–Glutathione Cycle Activity 

**Figure 6:**
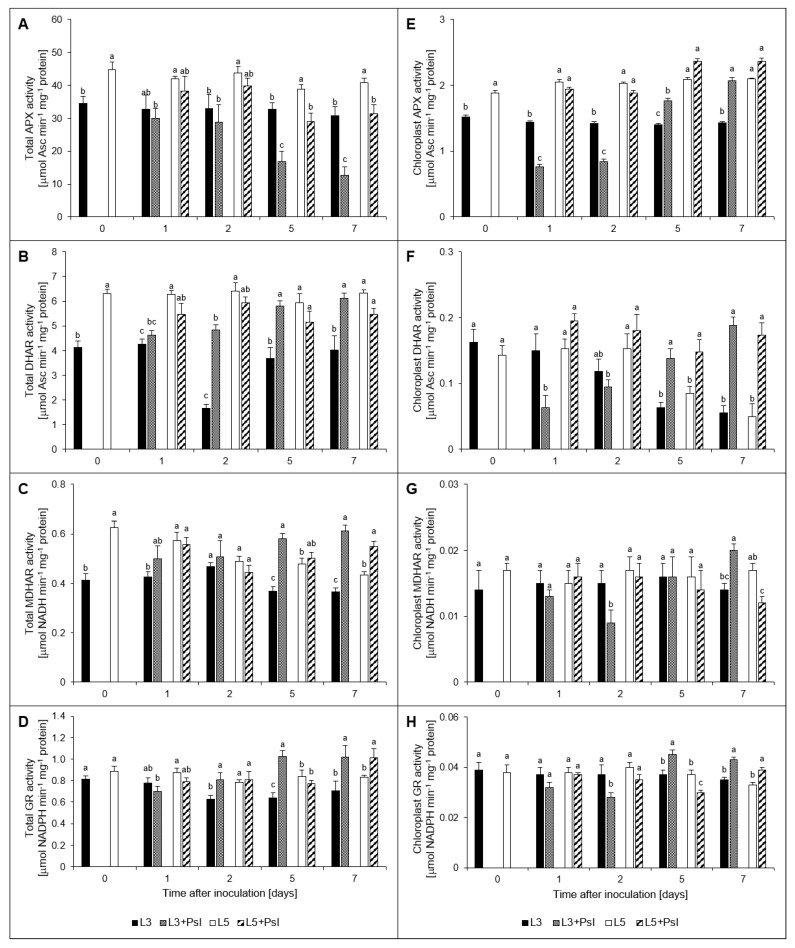
Ascorbate–glutathione cycle enzymes activities. Total activities (**A**–**D**) and chloroplast enzymes activities (**E**–**H**) were measured; (**A**,**E**) ascorbate peroxidase (APX) activities; (**B**,**F**) dehydroascorbate reductase (DHAR) activities; (**C**,**G**) monodehydroascorbate reductase (MDHAR) activities; (**D**,**H**) glutathione reductase (GR) activities. Values are means of four replicates (±SD). Different letters (a,b) indicate significant (*p* ≤ 0.05) differences between experimental variants within a given time point.

In the whole-leaf extract, the activity of APX constantly decreased after infection although this effect was more pronounced in the infected 3rd leaves than in the 5th ones. The strongest APX activity decline, to the level of 41% of the control, was found in the 3rd leaf at 7 dai (Figure 6A). Contrary to the changes observed for APX, *Psl* inoculation led to a noticeable increase in the activities of DHAR, MDHAR and GR in the 3rd leaf starting from the 2 dai (GR) or 5 dai (DHAR and MDHAR). At the advanced stage of infection symptoms development (5 and 7 dai), the activities of DHAR, MDHAR and GR in the 3rd leaves were induced to the levels ranging from 44% to 68% of the respective controls. The profile of *Psl*-induced DHAR, MDHAR, and GR activity changes in the 5th leaves did not match those described for the infected ones. The total activity of DHAR in the 5th leaves of the infected plants was decreased throughout the experiment while MDHAR and GR activities were increased only at 7 dai, up to 127% and 122% of the control levels, respectively (Figure 6C,E,G). 

The activities of all ascorbate–glutathione cycle enzymes in cucumber chloroplasts isolated from the 3rd leaves showed a biphasic pattern of infection-induced changes. The initial strong activity decrease (1–2 dai) was followed by an increase above the control level on the 5 dai and 7 dai (Figure 6B,D,F,H). The intensities of changes were specific for each enzyme. For example, a twofold decrease in APX activity in 3rd leaves observed at 1 dai and 2 dai was followed by activity increase in these leaves of 26% and 45% at 5 dai and 7 dai, respectively compared to the control. As to MDHAR, significant activity changes in chloroplasts of the infected 3rd leaves were found only at 2 dai (60% of the control level) and 7 dai (143% of control). Thus, at the advanced stage of infection symptoms development, i.e., at 7 dai, the activities of DHAR, MDHAR, and GR which are responsible for maintaining ascorbate and glutathione in the reduced forms, were significantly increased both in the whole leaf extracts and in chloroplasts of the infected leaves. Infection-induced changes in chloroplasts of the 5th leaves were observed at the advanced stage of disease symptom development only. For DHAR, they mirrored those described in chloroplasts isolated from the 3rd leaves. MDHAR activity in chloroplasts of the 5th leaves of infected plants was diminished by 29% at 7 dai when compared to the control whereas GR was decreased at 5 dai and enhanced at 7 dai, each time by about 19% (Figure 6F,H). 

**Figure 7:**
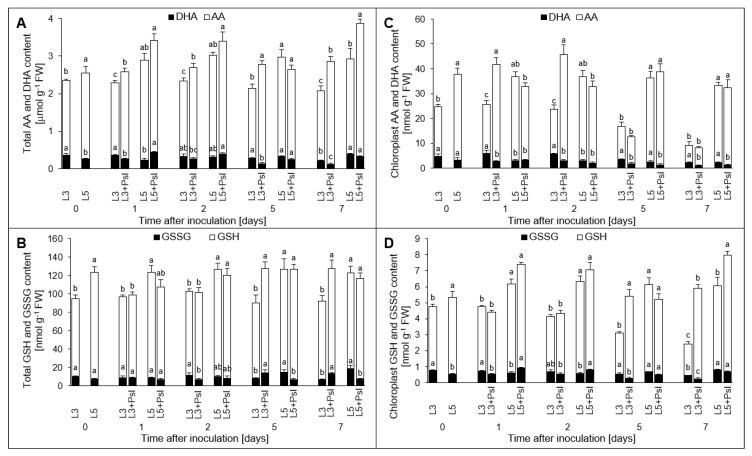
Ascorbate and glutathione contents. Total (**A**,**B**) and chloroplast (**C**,**D**) antioxidants contents were examined; (**A**,**C**) reduced ascorbate (AA) and oxidized ascorbate (dehydroascorbate, DHA); (**B**,**D**) reduced glutathione (GSH) and oxidized glutathione (glutathione disulfide, GSSG). Values are means of four replicates (±SD). Different letters (a–c) indicate significant (*p* ≤ 0.05) differences between experimental variants within a given time point. Ascorbate and glutathione contents were calculated on fresh weight basis because *Psl* infection did not change relative water content of leaves and, consequently, the fresh weight to dry weight ratio.

In the whole leaf extracts of control plants, the AA and GSH levels were continuously higher in the 5th leaves than in the 3rd ones (Figure 7A,C). The pattern of *Psl*-induced changes in AA content was roughly similar in the 3rd and 5th leaves. Its level in the whole leaf extracts constantly increased in the infected plants although significant changes were visible mainly in the 3rd leaves and in the 5th leaves at 7 dai. Infection brought about a strong DHA content decline in the 3rd leaf at 5 dai and 7 dai. This was concomitant with the induction of MDHAR and DHAR. However, in the 5th leaves the DHA concentration changes were observed at 1 dai and 5 dai when it increased up to 191% of the control and declined down to 76% of the control, respectively. The AA:DHA redox ratio in the whole leaf extract of control plants was maintained substantially higher in the 5th leaves than in the 3rd ones, except for 7 dai. Infection induced a prolonged AA:DHA redox ratio increase in the 3rd leaves. At the initial stage of disease symptom development, it was related to the accumulation of AA content whereas at 5 dai and 7 dai it also resulted from the decrease in DHA level. In the 5th leaves of infected plants, a significantly higher AA:DHA redox ratio was observed 7 dai whereas at 2 dai it was 41% lower than in the control (Table A2). 

In the whole leaf extracts, the infection-induced changes in the glutathione pool were manifested mainly at 5 dai and 7 dai by the increased GSH and GSSG contents in the 3rd leaves and the decreased GSSG content in the 5th ones (Figure 7C). Much like the relationship described for ascorbate, GSH:GSSG redox ratio in the whole leaf extract of control plants was maintained higher in the 5th leaves than in the 3rd ones, except for 5 and 7 dai. In the infected 3rd leaves, it remained significantly higher than in the control at 5 and 7 dai. However, in the 5th leaves of the infected plants the GSH:GSSG ratio started to rise reaching a significantly higher level than in the control plants at 5 and 7 dai (Table A2). 

In chloroplasts, the changes in AA content in the infected 3rd leaves showed an opposite trend when compared to that of APX. The concentration of AA significantly increased at 1 dai and 2 dai reaching 198% and 235% of the control, respectively and thereafter it tended to decrease (Figure 7B). Conversely, a long-lasting and pronounced decrease in DHA content was observed in the infected 3rd leaves. There was no effect of *Psl* infection on the concentration of chloroplastic AA in the 5th leaves whereas DHA content decrease was observed from 5 dai onward. The infection-induced perturbations in the chloroplastic AA and DHA pools drove concomitant changes in the ascorbate redox ratio (AA:DHA) which was significantly higher in the infected 3rd leaves in comparison to the control. Similar relationship was observed in the 5th leaves starting from the 2 dai (Table A2). Compared to AA, a reversed profile of infection-induced changes was found for chloroplastic GSH content in the infected 3rd leaves. Its significant accumulation at 5 and 7 dai could compensate for AA which concentration was diminished at these time points. The GSH content increase was accompanied by GSSG concentration decline to the level about 50% lower than in the control (Figure 7D). In consequence, at the advanced stages of disease symptom development, at 5 and 7 dai, the strongest chloroplastic GSH:GSSG redox ratio increases in the infected 3rd leaves were observed, up to the level 4-fold and 5-fold higher than in the control, respectively. The only significant change in the glutathione pool in chloroplasts from the 5th leaves of infected plants was visible for GSH at 7 dai, when its content was 39% higher than the control and hence the GSH:GSSG redox ratio was also 66% higher (Table A2). Apart from the pathogen-induced changes, the chloroplastic APX activity was leaf age-dependent and it remained significantly higher in the 5th leaves than in the 3rd ones, both in the infected and non-infected plants. This relationship was not observed for the other enzymes. However, it was found for AA and GSH contents as well as for AA:DHA and GSH:GSSG redox ratios (Figure 6 and Figure 7). 

### 2.5. Tocopherol Content

The total tocopherol content was markedly higher in the infected 3rd leaves than in the 5th ones. Both the 3rd and the 5th leaves preferentially accumulated α-tocopherol but in the 3rd leaves the tocopherol composition was shifted toward an increased γ-tocopherol content. In the 5th leaves, the content of δ-tocopherol was below detection limit at 0 and 2 dai and in the inoculated ones at 7 dai (Table 1). In the infected 3rd leaves a massive accumulation of tocopherol occurred at 2 dai, when the content of α-, γ-, and δ-tocopherol increased 4-, 3.4-, and 2-fold relative to control, respectively. However, at 7 dai the total tocopherol level was decreased and the content of α- and γ tocopherol was reduced by 31% and 19%, respectively. In the 5th leaves of the inoculated plants, due to the accumulation of α-tocopherol, the total tocopherol content was increased at 2 dai while at 7 dai it was decreased when compared to the uninoculated plants. At this time point, the content of α-tocopherol was reduced by 73% and it of γ-tocopherol—by 85% (Table 1). It is worth noting that the tocopherol composition in the 3rd and 5th leaves of the inoculated plants was significantly different as the 3rd leaves accumulated higher amounts of γ- and δ-tocopherol.

### 2.6. Gene Expression Analysis

**Figure 8:**
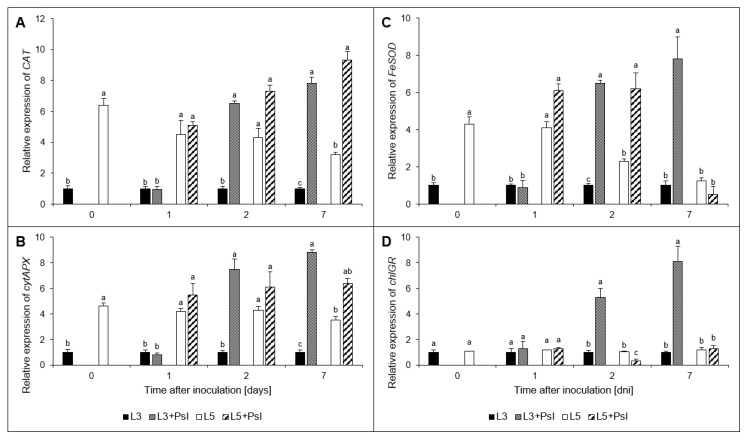
Relative expressions of antioxidant genes: (**A**) Catalase gene (*CAT*); **(B**) cytosolic ascorbate peroxidase gene (*cytAPX*); (**C**) chloroplast superoxide dismutase isoform gene (*FeSOD*); (**D**) chloroplast glutathione reductase gene (*chlGR*) expressions. Values are means of four replicates (±SD). Different letters (a–c) indicate significant (*p* ≤ 0.05) differences between experimental variants within a given time point.

At the local level, i.e., in the infected 3rd leaves, biotic stress increased the expression of *CAT*, *cytAPX*, *FeSOD,* and *chlGR* genes at 2 and 5 dai (Figure 8). They were from 4 to 8-fold increased relative to control. At the systemic level, i.e., in the 5th leaves of the infected plants, *CAT*, *cytAPX*, and *chlGR* were upregulated only at 5 dai, when compared to the 5th leaves of the control plants. The strongest stimulation, to the level 9-fold higher than in the control, was found for *chlGR.* In these leaves, *FeSOD* expression remained roughly unchanged when compared to the control.

## 3. Discussion

### 3.1. Differential Local and Systemic Responses of Photosynthesis to Psl Infection

Chloroplasts have recently emerged as central hubs for ROS/redox signaling which contribute to the outcome of plant–pathogen interactions [21,25]. Therefore, our understanding of how the photosynthesis-related information feeds into defense pathways will be highly relevant in attempts to simultaneously increase crop productivity and resistance to pathogens.

Pathogen infection interferes with photosynthesis in different ways. Chlorotic or necrotic lesions which are typical infection symptoms on leaves, reduce the photosynthetically active area [37]. Pathogen development leads to degradation of chloroplast structure [38] and toxins produced by pathogens increase chlorophyll degradation and modulate the chloroplast ROS balance to promote disease development [20,39]. Biotic stress usually downregulates photosynthesis although the mechanism and the nature of changes depend on the plant–pathogen interaction [19,40]. Reducing photosynthesis was suggested an effective defense mechanism against biotrophic/hemibiotrophic pathogens as it limits nutrient supply and restricts their survival on the host plant. Pathogens can modify this host response to their advantage. For example, *Xanthomonas citri* pv *citri* which causes citrus canker, produces a plant natriuretic peptide-like protein counteracting the repression of photosynthesis during infection and in that way the pathogen maintains the plant tissue in better condition for prolonged colonization [41]. Moreover, photosynthesis in the infected tissues must be switched off to initiate increased respiration and defense processes requiring energy, reducing equivalents and carbon skeletons [42]. In our study, *Psl* infection led to altered stomatal behavior, reduced chlorophyll autofluorescence, transpiration rate and the intracellular CO_2_ concentration in the inoculated 3rd cucumber leaves at a later stage of pathogenesis. *Psl* also induced a unique fluorescence signature characterized by a prolonged reduction in PSII efficiency in the 3rd leaves preceded by an increase in NPQ at 1 and 2 dai. All these interconnected events were observed in the 3rd leaves but not in the 5th leaves of the inoculated plants and could be responsible for reduced net photosynthesis rate becoming significant in the 3rd leaves with the expression of necrotic disease symptoms at 7 dai. Our results support a clear correlation between declined net photosynthesis rate and infection development described for other plant interactions with biotrophic pathogens [42,43]. However, the reduction in PSII efficiency and net photosynthesis rate in the 3rd leaves at 7 dai could be also attributable to senescence of the infected organ induced by hemibiotrophs as they switch from biotrophic to necrotrophic lifestyle [44].

The differential response of photosynthesis in the 3rd and the 5th cucumber leaves upon *Psl* infection could contribute to the whole plant functioning under biotic stress. The *Psl*-colonized 3rd leaves were source organs, and hemibiotrophic pathogen-infected source organs are usually subjected to a source-to-sink transition [45]. Thus, the higher maximal PSII quantum yield and net photosynthesis rate in the systemic leaves could support the increased demand for photoassimilates generated by the infected ones. Unchanged photosynthetic capacity in the 5th leaves could be important in providing energy for activating acclimation mechanisms or systemic defense responses as well as maintaining homeostasis and growth of the infected plant. Likely, the lower values of NPQ in the 5th leaves of inoculated plants at 1 and 2 dai reflect a greater ATP demand for systemic metabolic alterations, thus resulting in lower trans-thylakoid proton gradient which is one of the factors determining NPQ [46]. Changed NPQ level in the 5th leaves could also be an integral part of systemic stress signaling, as tissues along the path of the signals have been shown to have alternating NPQ levels [47]. In the inoculated 3rd leaves, however, NPQ which is negatively correlated with ROS production under excess excitation energy [48], could be involved in local plant defense mechanisms. Several studies have shown that the intensity of NPQ was negatively or positively affected by pathogens depending on the stage of pathogenesis and the type of interaction [49,50], and NPQ changes were caused by pathogen recognition per se and not by infection-associated stomata closure [22]. In *Arabidopsis* infected with *P. syringae* pv *tomato*, enhanced NPQ, followed by down-regulation of photosynthesis, was linked to facilitated infection due to suppression of chloroplastic ROS burst critical for the induction of PTI [51]. A similar tendency was observed in our plant–pathogen interaction in the infected 3rd leaves. 

### 3.2. Infection-Induced Local Changes in the Antioxidant System

Infection-induced imbalance in photosynthetic electron transport manifested by changes in the photochemical efficiency of PSII and NPQ in the 3rd leaves may initiate ROS and redox-linked signaling [52] and antioxidants in the infected cells play important role in transmitting the photosynthesis-derived signals [33]. The ascorbate–glutathione cycle, operating in all cellular compartments, has a key role in managing ROS generated under biotic stress and in redox signaling. In co-operation with SOD and CAT, the ascorbate–glutathione cycle strongly influences the compartment-specific ROS signature and owing to redox-based communication with other signaling pathways, it significantly contributes to the complex mechanism regulating plant stress response [6,14]. The activities of ROS-processing enzymes were differentially affected by the pathogen in the inoculated 3rd leaves. A general up-regulation of SOD, CAT, and ascorbate–glutathione cycle enzyme activities was found both for the whole-leaf extract and chloroplasts at the later stage of infection development (5–7 dai), except for APX which total activity decreased although the expression of *cytAPX* was increased. The accumulation of transcripts for cytosolic APX could reflect its involvement in ROS-related signal transmission between the chloroplast and the nucleus, and inhibition of APX by H_2_O_2_ was suggested to facilitate this signaling via micro-bursts [53,54]. 

Unlike the activities of H_2_O_2_-managing enzymes, total SOD activity was increased starting from the 1 dai. This could be the effect of infection-induced up-regulation of oxidative processes generating O_2_^·−^ in different compartments, especially in the apoplast which is the major site of extracellular ROS production in plant–pathogen interactions. The O_2_^−^ produced following the recognition of pathogen- and damage-associated molecular patterns undergoes dismutation to H_2_O_2_ which is used in cell wall strengthening processes or can function as a signaling molecule [55]. Interestingly, the earlier increase in total SOD activity was attributable to MnSOD and FeSOD whereas CuZnSOD and FeSOD were responsible for that at 7 dai. Comparing the activities of different subcellular isoforms of SOD suggests that *Psl* infection resulted in compartmentalized O_2_^−^ formation in the 3rd leaves which is an important determinant of ROS signal specificity [56], and chloroplasts were involved in the early and late cell responses as shown by the prolonged activation of FeSOD at the activity and mRNA levels. 

The mobilization of enzymatic ROS processing mechanisms in the 3rd leaves at the late stage of infection was supported by ascorbate and glutathione which contents were significantly increased in the whole-leaf extracts. Moreover, DHA was efficiently recycled to AA by the ascorbate–glutathione cycle enzymes and it was a remarkable shift of the ascorbate redox ratio toward the reduced state, as shown by the increase in the AA/DHA ratio. GSSG accumulation, which was rather due to increased DHAR activity than insufficient GR activity, led to GSH/GSSG ratio decrease. This indicated that the whole-leaf antioxidant response and redox buffering was preferentially linked to ascorbate, the major soluble antioxidant in the photosynthesizing cells, whereas more subtle and diverse changes in the glutathione redox ratio could be involved in stress signaling. As shown in other studies, the pathogen-induced changes in the ascorbate and glutathione pools are not necessarily parallel ensuring the specificity of redox signals mediated by these redox couples [14].

In chloroplasts of the 3rd leaves, the induction of the ascorbate–glutathione cycle enzymes at 5 and 7 dai potentially curtails the pathogen-induced oxidative burden during the advanced stage of infection, when limitations of CO_2_ supply may cause chloroplasts to produce ROS via O_2_ photoreduction. Their earlier activity decreases promoting H_2_O_2_ accumulation, fit into the concept of the critical role of chloroplastic ROS burst, particularly signals generated by H_2_O_2_ in chloroplasts, in inducing immune responses [57,58]. However, this could not be the case in our study as at that time the chloroplast redox environment appeared to be shifted toward the reduced state due to increased AA content and AA/DHA redox ratio. We also found a specific link between APX activity and the AA content, as decreased APX activity was related to increased AA content and vice versa. Moreover, a strong positive correlation between AA and tocopherol was observed. Tocopherols, mainly the α and γ homologues, in a coordinated action with AA and GSH, help to protect the photosynthetic membranes from lipid peroxidation, scavenge singlet molecular oxygen (^1^O_2_) and maintain the functioning of the photosynthetic apparatus under stress [59]. It has also been suggested that altered content and composition of the tocopherol pool influence interactions with pathogens [60]. We observed that at 2 dai, *Psl* infection induced the accumulation of α-tocopherol and γ-tocopherol which is the precursor of α-tocopherol. This coordinated elevation of tocopherols and AA may increase the tocopherol recycling capacity, enhance the ability of chloroplasts to scavenge ROS and to avoid oxidative damage at an earlier stage of infection. Changes in α-tocopherol and γ-tocopherol contents in the 3rd inoculated leaves were biphasic. As in other plants exposed to stress [61], an initial increase at 2 dai was followed by net total tocopherol loss at 7 dai, and these changes mirrored those for AA. It has been suggested that tocopherol content tends to decrease as the oxidative stress is more severe and ROS generation in chloroplasts increases [62]. As light-dependent stimulation of AA biosynthesis requires photosynthetic electron transport activity [63], AA accumulated in chloroplasts only in parallel to increase in photochemical quenching (Qp) and NPQ, prior to potential H_2_O_2_ overproduction, and not when the maximal quantum yield decreased (at 5 and 7 dai). In chloroplasts of the 3rd leaves, the decrease in the ascorbate pool was visibly compensated by glutathione accumulation. This co-operation between tocopherol, ascorbate and glutathione maintains the redox buffering and is essential in cyclic redox reactions [64]. It could also determine the temporal specificity of chloroplast-derived redox signals contributing to local plant response to *Psl* infection and systemic signaling. The activation of antioxidant mechanisms, however, was not sufficient to prevent oxidative damage of PSII. At the advanced stage of infection, lower *F_v_*/*F_m_* values were observed and NPQ which protects from ROS, mainly ^1^O_2_ responsible for oxidative injury [65], was not induced. Together with the factors limiting CO_2_ assimilation, this led to net photosynthesis rate decline in the 3rd inoculated leaves.

### 3.3. Systemic Antioxidant Response to Local Infection

ROS and cognate redox signals are involved in systemic signaling networks that allow the information of local stress to be propagated and to elicit whole plant response [49,66]. Systemic signaling has been extensively studied during pathogen-induced systemic acquired resistance (SAR) and plant acclimation response to abiotic stress [67,68]. In our study, localized infection restricted to the inoculated 3rd leaves, triggered long-range signaling translated into specific redox signature in the systemic 5th leaves. The local and systemic antioxidant responses shared some components, but the specific redox regulations were distinct. Only the activation of catalase, which is a kind of housekeeping enzyme involved mainly in photorespiratory H_2_O_2_ scavenging [69], was similar in the 3rd and 5th leaves at both activity and transcription levels. In general, changes in the antioxidant system in the inoculated 3rd leaves were initiated earlier and reached higher intensity than those in the systemic ones. For whole-leaf extracts, only MDHAR and GR activity increases were like those observed in the 3rd inoculated leaves, at least at 7 dai. In the 5th leaves of the inoculated plants, both ascorbate and glutathione pools underwent a general shift towards the reduced state at 5 and 7 dai, as shown by high AA/DHA and GSH/GSSG ratios. This GSH-related redox signature was the opposite of that in the 3rd inoculated leaves. These whole-leaf changes in the 5th leaves could be viewed as an attempt to maintain a dynamic redox balance of the ascorbate and glutathione pools in the cytoplasm which is the main cellular reservoir of these antioxidants [70,71]. Due to this high antioxidant capacity, the cytoplasm is envisaged as a buffer zone maintaining low concentration of ROS used for signaling [72].

In the systemic leaves, the ascorbate–glutathione cycle constituents generated chloroplast-specific changes which contrasted with the response of locally infected leaves, except for DHAR induction at 5 and 7 dai. Whatever the details of the underlying infection-induced mechanisms related with ascorbate–glutathione cycle activity and tocopherol content, the chloroplast pool of ascorbate in the systemic leaves was more reduced, while that of glutathione was in less reduced state than in the inoculated 3rd leaves, and the composition of tocopherol pool was altered. Besides its role as an antioxidant, glutathione is extensively linked to the regulation of defense against pathogens, i.e., through redox signaling including the activation of NPR1 in SA-mediated gene expression, and biosynthesis of S-containing defensive secondary metabolites [71]. Moreover, the redox state of glutathione regulates translation of Rubisco large subunit in chloroplasts [73]. We suggest that the changes in the ascorbate- and glutathione-related redox signature in the systemic leaves induced by local bacterial infection, linked to the modified photochemical activity, could represent redox homeostasis maintaining mechanism which allows controlling gene expression and metabolic activities, thus supporting the defensive capacity and growth of the infected plant. They may also determine a priming response of systemic leaves to future infection [74,75]. 

## 4. Material and Methods

### 4.1. Biological Material

**Figure A2:**
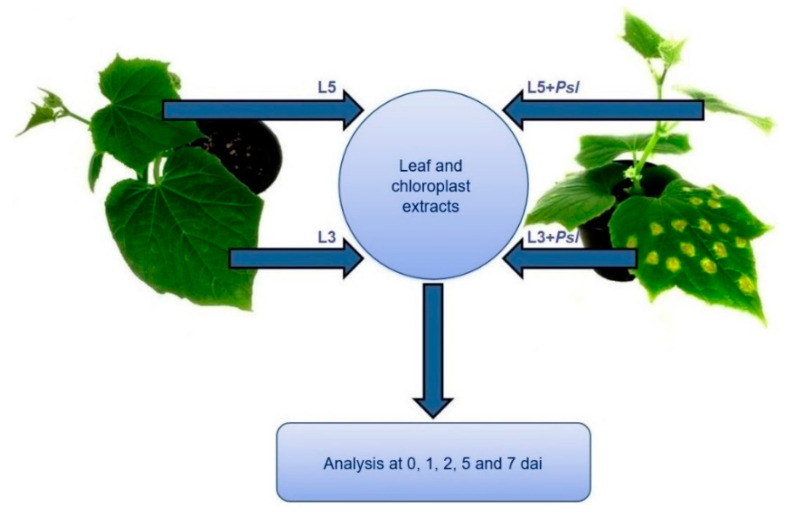
The experimental model. The 3rd leaves were inoculated with *Psl* (L3 + *Psl*) or treated with sterile distilled water (control, L3). The 5th leaves were non-inoculated and collected from control (L5) or inoculated plants (L5 + *Psl*). Leaves and isolated chloroplasts were taken for analyses on 0, 1, 2, 5, and 7 dai after inoculation.

Cucumber (*Cucumis sativus* L.) cv Cezar) plants were grown in a growth chamber, in soil at 25 °C with 16/8 h (day/night) photoperiod and 350 μmol m^−2^ s^−1^ photosynthetic photon flux density. Four-week-old plants were inoculated with *Psl* (isolate No. IOR 1990, Bank of Plant Pathogens, Poznań, Poland). Third true leaves of cucumber plants were inoculated with *Psl* suspension (10^7^ CFU cm^−3^) or treated with sterile distilled water (control) using a needle-less hypodermic syringe [6]. Inoculated (3rd) and non-inoculated (5th) leaves were taken for analyses on 0, 1, 2, 5, and 7 dai after inoculation (day) (Figure A2).

### 4.2. Infrared Thermography and Fluorescence Microscopy 

The visualization of the inoculated (3rd) leaves temperature profiles was performed 2 and 7 dai by using a high resolution (320 × 256 pixels) infrared camera FLIR E50 (FLIR Systems, Inc., Wilsonville, OR, USA) with a spectral range of 3.5–5.0 m and a sensitivity of 0.07 °C and showed in the form of pseudocoloured infrared images. The change in chlorophyll fluorescence in the inoculated leaf areas was observed under Nikon ECLIPSE Ni epifluorescence microscope (Nikon, Tokyo, Japan) equipped with Microscope Camera Digital Sight series DS-Fi1c and NIS Imaging, Nikon v. 4.11 software. 

### 4.3. Chlorophyll Fluorescence and Gas Exchange Analyses

Chlorophyll *a* fluorescence was measured in leaves by using FluorCAM imaging system (PSI, Brno, Czech Republic). Minimum (*F_o_*) and maximum (*F_m_*) chlorophyll *a* fluorescence as well as light-adapted fluorescence parameters (*F_o_*^’^ and *F_m_*^’^) were measured. The maximal PSII quantum yield (*F_v_/F_m_*), non-photochemical quenching (NPQ) as well as the coefficient of photochemical quenching (*Q_P_*) were calculated according to Nosek et al. [76]. Measurements were performed according to the manufacturer’s instruction.

Intracellular carbon dioxide concentration (*C_i_*), transpiration rate (*E*), stomatal conductance (G*s*) and net photosynthesis rate (*P_N_*) were determined using an infrared gas analyzer CIRAS 2 with the 2.5 cm^2^ PLC 4 Board (PP Systems, Hitchin, UK). The gas exchange intensity was set out in the air containing 21% of O_2_ and 375 μmol CO_2_ mol^−1^ at 25 °C and with 350 μmol m^−2^ s^−1^ photosynthetic photon flux density in a closed system with 300 cm^3^ min^−1^ flow through the measuring cell. As shown earlier, *Psl* infection did not change the leaf relative water content [6].

### 4.4. Chloroplasts Isolation

To isolate chloroplasts, 5.0 g of fresh 3rd and 5th cucumber leaves without the midribs were homogenized in 50 mM Tris-HCl buffer containing 10 mM Na_4_P_2_O_7_, 5 mM MgCl_2_, 1 mM dithiothreitol (DTT), 0.3 M sorbitol and 0.05% bovine serum albumin (BSA), pH 6.5. The homogenate was filtered through one layer of Miracloth (Merck Millipore, Burlington, MA, USA) and centrifuged (4 °C, 5 min, 780× *g*). The pellet was resuspended in 1 cm^3^ of 50 mM Tris-HCl buffer containing 0.3 M sorbitol, pH 7.0 (resuspension buffer), applied to 50% Percoll (Sigma Aldrich, St. Louis, MO, USA) gradient and centrifuged (4 °C, 8 min, 780× *g*). The resulting chloroplast suspension was washed three times and resuspended in 0.5 cm^3^ of the resuspension buffer. The intactness of chloroplasts was evaluated microscopically and determined with the ferricyanide method [77]. Purity of the chloroplast fraction was monitored by analysis of activities of glucose-6-phosphate dehydrogenase (EC 1.1.1.49) [78], fumarase (EC 4.2.1.2) [79], and catalase (EC 1.11.1.6) [80] as marker enzymes for cytosol, mitochondria and peroxisomes, respectively.

### 4.5. Determination of SOD Activity and SOD Native PAGE Electrophoresis

For determination of SOD (EC 1.15.1.1), 0.5 g of the 3rd and 5th leaves or 0.5 cm^3^ of the chloroplast suspension were homogenized in 2.5 cm^3^ or 1.0 cm^3^, respectively of 50 mM sodium phosphate buffer containing 0.1 mM ethylenediamine-tetraacetic acid tetrasodium salt (EDTA), 1% polyvinyl-pyrrolidone (PVP) and 1 M NaCl, pH 7.8. After centrifugation (4 °C, 20 min, 20,000× *g*) the supernatants were taken for SOD activity determination according to the method of Beauchamp and Fridovich [81] which measures the inhibition of photochemical reduction of NBT. One unit of SOD activity was defined as the 50% decrease of SOD-inhibitable NBT reduction.

For SOD electrophoresis, 0.5 g of the 3rd and 5th leaves without the midribs were homogenized in 2.5 cm^3^ of 50 mM Tris-HCl buffer containing 3 mM EDTA, 1 mM MgCl_2_ and 2% PVP, pH 8.0. After centrifugation (4 °C, 20 min, 20,000× *g*) plant extract containing 20 μg of protein supplemented with 0.01% bromophenol blue and 40% sucrose (2:1) was subjected to discontinuous PAGE under nondenaturing conditions, at 4 °C, 180 V, using 10 mM Tris-HCL buffer pH 8.3, containing 80 mM glycine without sodium dodecyl sulphate, in 7.5% polyacrylamide gel, with a constant current of 30 mA per gel. Identification of SOD isoforms was achieved by preincubation of the gel in 50 mM sodium phosphate buffer, pH 7.0 containing 3 mM KCN (CuZn-SOD inhibitor) and 5 mM H_2_O_2_ (CuZn-SOD and Fe-SOD inhibitor) for 30 min. After preincubation, the gel was incubated for 25 min in 50 mM sodium phosphate buffer, pH 7.8 containing 2.5 mM NBT and then in 50 mM sodium phosphate buffer, pH 7.8 containing 28 μM riboflavin and 28 μM *N*,*N*,*N*′,*N*′-tetramethyletylenediamine (TEMED, Sigma Aldrich, St. Louis, MO, USA), under darkness for 20 min [81]. Visualization of SOD bands was performed under daylight for 20 min [82].

### 4.6. Determination of CAT and the Ascorbate–Glutathione Cycle Enzymes Activities

For determination of APX (EC 1.11.1.11), DHAR (EC 1.8.5.1), MDHAR (EC 1.6.5.4) and GR (EC 1.8.1.7), 0.5 g of the 3rd and 5th leaves or 0.5 cm^3^ of the chloroplast suspension were homogenized in 2.5 cm^3^ or 1.0 cm^3^, respectively of 50 mM sodium phosphate buffer containing 0.1 mM EDTA, 1% PVP and 1 M NaCl, pH 7.5, supplemented with 1 mM sodium ascorbate or 0.02% mercaptoethanol for APX and DHAR activity determination, respectively. After centrifugation (4 °C, 20 min, 20,000 × *g*) the supernatant was used for determination of enzyme activities. CAT activity was assayed in the whole-leaf extracts prepared as above, by measuring H_2_O_2_ decomposition (ε = 36.0 mM^−1^ cm^−1^) according to Dhindsa et al. [80]. APX, DHAR, MDHAR and GR activities were assayed as described earlier [14]. APX activity was measured as a rate of H_2_O_2_-dependent oxidation of ascorbate (ε = 13.7 mM^−1^ cm^−1^) to dehydroascorbate. DHAR activity was determined as a rate of glutathione-dependent formation of ascorbate (ε = 13.7 mM^−1^ cm^−1^). MDHAR activity was assayed as a rate of monodehydroascorbate-dependent oxidation of NADPH (ε = 6.22 mM^−1^ cm^−1^). GR activity was determined by following glutathione disulfide-dependent oxidation of NADPH. The activities of APX, MDHAR, DHAR, and GR were given in μmol min^−1^ mg^−1^ protein. 

### 4.7. Determination of Protein Content

Protein concentration in the chloroplast extract was determined spectrophotometrically, according to Bradford [83].

### 4.8. Determination of Ascorbate and Glutathione Contents

For determination of reduced ascorbate (AA) and dehydroascorbate (DHA) contents, 0.5 g of the 3rd and 5th leaves or 0.5 cm^3^ of the chloroplast suspension were homogenized in 2.5 cm^3^ or 0.75 cm^3^, respectively of 10% trichloroacetic acid (TCA). Ascorbate was determined according to the colorimetric 2,2′-bipyridyl method [84]. Total ascorbate (AA + DHA) content was assayed by adding DTT to reduce DHA to AA in the samples. DHA content was calculated by subtracting the AA content from the total ascorbate. Concentrations of AA, DHA and the total ascorbate were given in μmol g^−1^ fresh weight (FW) for whole leaves or in nmol g^−1^ FW for chloroplasts and evaluated using the standard curve prepared for AA.

For determination of reduced glutathione (GSH) and glutathione disulfide (GSSG) contents, 0.5 g of the 3rd and 5th leaves or 0.5 cm^3^ of the chloroplast suspension were homogenized in 2.5 cm^3^ or 0.75 cm^3^, respectively of 0.05 M sodium phosphate buffer, pH 6.5. Glutathione was determined by the modified 5,5′-dithiobis-(2-nitrobenzoic) acid (DTNB) method [85]. GSSG content was determined by adding 2-vinylpyridine (Sigma Aldrich, St. Louis, MO, USA) to remove GSH. GSH concentration was calculated by subtracting the GSSG value from the total glutathione. The standard curve was prepared for GSH and glutathione content was given in nmol g^−1^ FW.

### 4.9. Gene Expression Analysis by Quantitative Real-Time PCR

Total RNA was extracted from cucumber leaves (50 mg) using GeneMATRIX Universal DNA/RNA/Protein Purification Kit (Eur_x_^®^, Gdańsk, Poland) following the manufacturer’s instruction. RNA was purified from a genomic DNA (6U DNAse I per a sample, Eur_x_^®^, Gdańsk, Poland) and flushed twice with using Wash Buffers RB1 and RBW according to the manufacturer’s instruction (GeneMATRIX Universal DNA/RNA/Protein Purification Kit, Eur_x_^®^, Gdańsk, Poland). The total RNA product was quantified by spectrophotometric assay using NanoDrop^®^ (ND-1000, Thermo Fisher Scientific, Wilmington, DE, USA). Absorbance ratios (A260/A280 and A260/A230) were measured and a value approximately of 2 was received in all samples indicating non-contamination of the RNA products. The evaluation of isolated RNA integrity was performed by the agarose gel electrophoresis and an intact of 18S and 28S rRNA was observed indicating the minimal RNA degradation. Thereafter, 800 ng of RNA was used for the reverse-transcription polymerase chains reaction. The cDNA was synthesized in the volume of 20 μL using NG dART RT kit (Eur_x_^®^, Gdańsk, Poland), according to the manufacturer’s instruction. Quantitative Real-time PCR assays were performed on the LightCycler 480 II (Roche Applied Science, Penzberg, Germany) using SensiFAST SYBR^®^ No-ROX kit (Bioline, London, United Kingdom). The qRT-PCR reactions were carried out in 15 μL of mixture containing 7.5 μL of 2× Master Mix SYBRGreen A^®^ (A&A Biotechnology, Gdynia, Poland), 2.5 μL of specific forward and reverse primers (3 μM, Table A3) and 5 μL of 15-times diluted cDNA preparations or water (negative control). The mixture of three randomly selected cDNA preparations was 4, 12, 36, 108, and 324-times diluted for the standard curve. The qRT-PCR reactions were performed with the following cycles: (1) an initial denaturation (95 °C, 5 min), (2) 35 cycles of amplification (95 °C, 10 s – 55 °C, 10 s – 72 °C, 10 s – the fluorescence measurement), (3) melting curve analysis (65–95 °C, 0.1 °C s^−1^), (4) cooling the machine (40 °C, 30 s). Relative quantification of qRT-PCR was carried out using Livak’s method. The C_T_ values of the target (CAT, Fe-SOD, cyt-APX, chl-GR) genes were normalized to the C_T_ values of the reference (α-TUB, UBI-ep) genes for both the test and the calibrator samples. Primers sequences for target and references genes are shown in Table A3. All calculations and statistical analyses were performed using Excel (MS Office 365^®^) and Statistica^®^ ver. 12.

### 4.10. Determination of Tocopherols by GC-MS

Leaf samples (1 g) were ground with liquid nitrogen and extracted for 30 min on a magnetic stirrer in 20 mL of n-hexane containing 0.05 mL of internal standard (n-hexane solution of n-docosane at the concentration of 1 mg mL^−1^). The extract was filtered through filter paper and the pellet was extracted twice into n-hexane without n-docosane. The extracts were pooled, dried under vacuum at 65 °C and resuspended in n-hexane.

Hexane extracts were analyzed with a HP 6890 Gas Chromatograph equipped with mass selective detector MSD 5973 (Agilent Technologies, Santa Clara, CA, USA) which was fitted with autosampler 7693A ALS system, electronic pressure control and split/splitless injector. The injector worked in a split 1:50 mode at 250 °C. Volume of sample introduced into injector was 1 μL. Transfer line temperature was 280 °C. Separation was performed on HP-5ms (30 m × 0.25 mm; 0.25 μm film thickness) fused silica column with helium flow rate 1 mL/min [86].

The EIMS spectra were obtained at 70 eV ionization energy, at the source temperature 230 °C and that of quadrupole 150 °C. The MSD was set to scan at 40–620 a.m.u. Chromatograms were registered in linear temperature programmed regime from 50 °C to 320 °C at the rate 3 °C /min [86].

**Figure A3:**
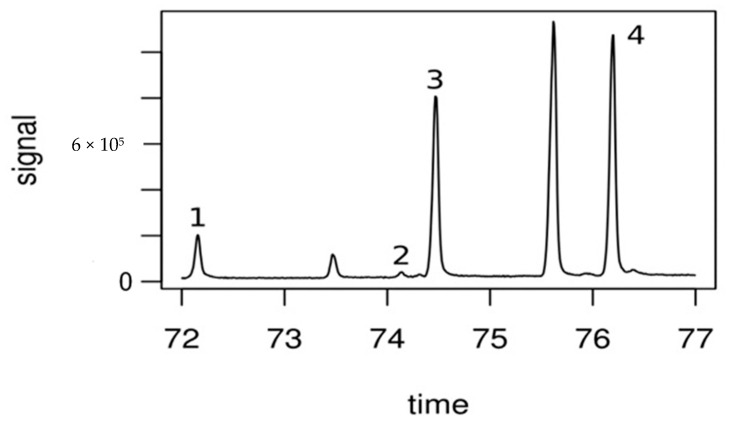
An exemplary GC-MS chromatogram of tocopherols. 1—d-tocopherol, 2—b-tocopherol, 3—g-tocopherol, 4—a-tocopherol. In GC–MS analyses, the identifications required two independent analytical parameters: Mass spectrum and retention index [87]. Therefore, a mixture of C_9_-C_40_ n-alkanes in n-hexane was used to calculate retention indices of chromatographic peaks. Experimental values of retention indices and mass spectra were compared with those from literature databases (NIST’11, Adams 4th), and the home-made database (L.S.).

For all the samples, chromatograms were integrated to obtain retention times and areas of chromatographic peaks using Chemstation software (Agilent Technologies, Santa Clara, CA, USA). An exemplary chromatogram with tocopherols is shown in Figure A3.

### 4.11. Statistical Analysis

Kruskal–Wallis test for statistical analysis of the results was performed using the Statistica^®^ software (ver. 12, StatSoft, Inc., Tulsa, OK, USA). We used the Kruskal–Wallis test followed by Dunn multiple comparison post-hoc since that data were non-parametric. The random variables in all the studied groups were not normally distributed. Moreover, variances of the studied groups were heteroscedastic. The data are means from 4 independent experiments and two plants for each experimental variant and time point were analyzed in each experiment. Sample variability is given as a standard deviation of arithmetic average. Differences at *p* ≤ 0.05 were considered as significant.

## 5. Conclusions

*Psl* infection strongly affected the cellular and chloroplast ROS/redox equilibrium in the infected leaves, as shown by changes in the activities of SOD, CAT, the ascorbate–glutathione cycle and in the tocopherol pool. However, the co-activation of antioxidant mechanisms was not sufficient to prevent oxidative damage of PSII. Together with the infection-related factors limiting CO_2_ assimilation, this led to photosynthesis inhibition in leaves locally infected by *Psl* at advanced stage of pathogenesis. The pathogen-induced decline in photochemical activity and net photosynthesis rate was restricted to the infected leaves whereas the systemic ones retained their photosynthetic capacity for potential activating acclimation and defense mechanisms. Local bacterial infection induced a systemic response visible by changes in the photochemical activity and the reorganization of the antioxidant system in the non-inoculates leaves. The interplay between ascorbate, glutathione and tocopherol could determine the specificity of redox signaling originating from chloroplasts of the infected leaves and the pathogen-free systemic ones. Local signals, potentially originating from pathogen-induced disturbances in the photochemical activity of photosynthesis and redox homeostasis, were translated into specific ascorbate-, glutathione-, and tocopherol-related redox signature, especially evident in chloroplasts of the systemic leaves, which could contribute to mechanisms regulating plant-wide responses. These results further emphasize the relation between ROS/redox signaling, photosynthesis, and biotic stress and the implication of chloroplasts in mediating local and systemic plant defense responses.

## Figures and Tables

**Table 1:** Tocopherols relative contents.

Time after Inoculation [days]	0		2				7			
Tocopherol	L3	L5	L3	L3 + *Psl*	L5	L5 + *Psl*	L3	L3 + *Psl*	L5	L5 + *Psl*
α-tocopherol	208.9 ± 53.9 (a)	115.1 ± 69.5 (a)	94.2 ± 10.9 (b)	199.4 ± 15.4 (a)	101.7 ± 25.3 (b)	138.3 ± 41.5 (ab)	222.5 ± 17.4 (b)	152.0 ± 1.1 (c)	373.7 ± 86.1 (a)	100.6 ± 11.2 (d)
ɣ-tocopherol	147.5 ± 48.8 (a)	17.9 ± 8.6 (b)	50.2 ± 5.6 (b)	171.9 ± 12.8 (a)	23.2 ± 7.6 (c)	18.9 ± 1.1 (c)	205.0 ± 14.0 (a)	165.7 ± 18.8 (b)	100.2 ± 28.9 (b)	14.6 ± 8.5 (c)
δ-tocopherol	13.7 ± 4.0 (a)	-	8.2 ± 4.0 (b)	33.0 ± 10.2 (a)	-	2.8 ± 1.2 (b)	59.3 ± 3.8 (a)	66.4 ± 3.1 (a)	2.8 ± 4.0 (b)	-
**Total**	370.1 ± 27.5 (a)	133.0 ± 31.8 (b)	152.6 ± 6.1 (b)	404.3 ± 11.8 (a)	124.9 ± 10.7 (b)	160.0 ± 18.6 (b)	486.8 ± 10.9 (a)	384.1 ± 12.7 (b)	476.7 ± 74.3 (a)	115.2 ± 9.7 (c)

Values are means of four replicates (±SD). Different letters (a–d) indicate indicate significant (*p* ≤ 0.05) differences between experimental variants within a given time point.

## Abbreviations

^1^O_2_	Singlet molecular oxygen
AA	Ascorbic acid reduced
APX	Ascorbate peroxidase
CAT	Catalase
C*_i_*	Intracellular carbon dioxide concentration
DHA	Dehydroascorbic acid
DHAR	Dehydroascorbic acid reductase
E	Transpiration rate
ETI	Effector-triggered immunity
Fv/Fm	Maximal PSII quantum yield
GR	Glutathione reductase
G*s*	Stomatal conductance
GSH	Glutathione reduced
GSSG	Glutathione disulfide
HR	Hypersensitive response
MAPK	Mitogen-activated protein kinase
MDHAR	Monodehydroascorbic acid reductase
NPQ	Non-photochemical quenching
NPR1	non-expressor of pathogenesis-related gene 1
O_2_^−^	Superoxide anion radical
P*_N_*	Net photosynthesis rate
PSII	Photosystem II
PTI	Pathogen-associated molecular patterns (PAMP)-triggered immunity
Q_P_	Photochemical quenching
ROS	Reactive oxygen species
SOD	Superoxide dismutase

## Appendix A

**Table A1:** Relative activities of superoxide dismutase (SOD) isoforms after native gel electrophoresis (PAGE).

Time after Inoculation [days]	Isoform	L3	L3 + *Psl*	L5	L5 + *Psl*
0	MnSOD-1	1.31 ± 0.23 (a)		1.25 ± 0.18 (a)	
	MnSOD-2	0.38 ± 0.04 (a)		0.19 ± 0.06 (b)	
	MnSOD-3	0.44 ± 0.02 (a)		0.22 ± 0.01 (b)	
	MnSOD-4	0.63 ± 0.03 (a)		0.16 ± 0.03 (b)	
	FeSOD	1.16 ± 0.12 (a)		0.88 ± 0.07 (a)	
	CuZnSOD-1	1.28 ± 0.07 (a)		1.22 ± 0.06 (a)	
	CuZnSOD-2	1.00 ± 0.09 (a)		0.88 ± 0.13 (a)	
2	MnSOD-1	1.26 ± 0.22 (a)	1.46 ± 0.11 (a)	1.31 ± 0.04 (a)	1.34 ± 0.22 (a)
	MnSOD-2	1.03 ± 0.07 (b)	1.40 ± 0.03 (a)	1.06 ± 0.07 (b)	1.00 ± 0.04 (b)
	MnSOD-3	0.83 ± 0.06 (b)	1.34 ± 0.08 (a)	0.89 ± 0.06 (b)	0.80 ± 0.06 (b)
	MnSOD-4	0.54 ± 0.01 (b)	1.34 ± 0.23 (a)	0.60 ± 0.07 (b)	0.63 ± 0.11 (b)
	FeSOD	1.86 ± 0.02 (a)	1.51 ± 0.04 (b)	2.06 ± 0.11 (a)	1.97 ± 0.08 (a)
	CuZnSOD-1	1.23 ± 0.18 (b)	1.37 ± 0.19 (b)	2.46 ± 0.13 (a)	2.34 ± 0.18 (a)
	CuZnSOD-2	1.00 ± 0.04 (b)	1.06 ± 0.03 (b)	1.51 ± 0.07 (a)	1.49 ± 0.15 (a)
7	MnSOD-1	1.45 ± 0.03 (a)	1.41 ± 0.23 (a)	1.48 ± 0.05 (a)	1.41 ± 0.03 (a)
	MnSOD-2	1.28 ± 0.14 (a)	1.38 ± 0.04 (a)	1.24 ± 0.11 (a)	1.10 ± 0.11 (a)
	MnSOD-3	1.21 ± 0.06 (a)	1.41 ± 0.16 (a)	1.17 ± 0.03 (a)	0.83 ± 0.05 (b)
	MnSOD-4	1.17 ± 0.11 (a)	1.45 ± 0.11 (a)	1.10 ± 0.07 (a)	0.38 ± 0.01 (b)
	FeSOD	1.69 ± 0.07 (b)	3.86 ± 0.09 (a)	1.76 ± 0.22 (b)	0.59 ± 0.06 (c)
	CuZnSOD-1	1.10 ± 0.09 (b)	3.14 ± 0.16 (a)	1.41 ± 0.14 (b)	1.34 ± 0.03 (b)
	CuZnSOD-2	1.00 ± 0.15 (b)	2.48 ± 0.06 (a)	0.97 ± 0.02 (b)	0.97 ± 0.07 (b)

Values are means of four replicates (±SD). Different letters (a–c) indicate significant (*p* ≤ 0.05) differences between experimental variants within a given time point.

**Table A2:** Redox ratios for ascorbate and glutathione defined as the quotient of reduced (AA, GSH) to oxidized (DHA, GSSG) content form in leaf (tot) and chloroplast (chl) extracts.

Redox Ratio	Time after Inoculation [days]	L3	L3 + *Psl*	L5	L5 + *Psl*
[AA]_tot_/[DHA]_tot_	0	5.5 ± 0.2 (b)		8.8 ± 0.4 (a)	
	1	5.4 ± 0.1 (b)	9.2 ± 0.6 (a)	11.6 ± 0.3 (a)	6.9 ± 0.5 (b)
	2	6.2 ± 0.3 (a)	9.3 ± 0.2 (a)	8.8 ± 0.8 (a)	7.6 ± 0.1 (a)
	5	.0 ± 0.6 (b)	20.3 ± 0.5 (a)	8.1 ± 0.4 (b)	9.7 ± 0.9 (b)
	7	8.7 ± 0.3 (b)	23.4 ± 0.8 (a)	6.5 ± 0.9 (b)	10.8 ± 1.0 (b)
[GSH]_tot_/[GSSG]_tot_	0	8.8 ± 0.5 (b)		15.4 ± 0.3 (a)	
	1	10.2 ± 1.2 (a)	10.9 ± 0.5 (a)	13.5 ± 0.8 (a)	14.9 ± 1.0 (a)
	2	7.9 ± 0.6 (b)	14.2 ± 0.4 (a)	11.9 ± 0.2 (a)	13.7 ± 0.3 (a)
	5	10.8 ± 0.4 (b)	8.0 ± 0.8 (b)	7.7 ± 0.7 (b)	18.8 ± 0.9 (a)
	7	13.4 ± 0.6 (a)	8.8 ± 0.3 (b)	5.7 ± 0.3 (c)	15.0 ± 0.7 (a)
[AA]_chl_/[DHA]_chl_	0	4.2 ± 0.6 (b)		10.8 ± 0.2 (a)	
	1	3.2 ± 0.3 (b)	13.4 ± 0.9 (a)	10.7 ± 0.7 (a)	9.1 ± 0.3 (a)
	2	3.2 ± 0.1 (b)	14.0 ± 0.8 (a)	11.1 ± 0.1 (a)	14.3 ± 0.5 (a)
	5	3.8 ± 0.8 (c)	5.6 ± 0.6 (c)	13.3 ± 0.3 (b)	25.7 ± 0.5 (a)
	7	3.0 ± 0.2(d)	6.3 ± 0.3 (c)	13.0 ± 0.8 (b)	23.9 ± 0.7 (a)
[GSH]_chl_/[GSSG]_chl_	0	5.3 ± 0.7 (b)		8.9 ± 0.3 (a)	
	1	5.4 ± 0.3 (b)	7.4 ± 0.2 (b)	9.3 ± 0.1 (a)	7.2 ± 0.2 (b)
	2	5.2 ± 0.7 (b)	7.0 ± 0.8 (b)	9.7 ± 0.2 (a)	7.7 ± 0.3 (b)
	5	4.7 ± 0.1 (c)	19.1 ± 0.3 (a)	8.2 ± 0.1 (b)	9.6 ± 0.8 (b)
	7	4.5 ± 0.6 (c)	26.5 ± 1.2 (a)	6.5 ± 0.9 (c)	10.8 ± 1.1 (b)

Values are means of four replicates (±SD). Different letters (a–c) indicate significant (*p* ≤ 0.05) differences between experimental variants within a given time point.

**Table A3:** Primer sequences for the target and reference genes.

NCBI Accesion Number	Forward Primer	Reverse Primer
GU248529 (*CAT*)	TTCCAATCAATTACCGTCACAT	ATTCTAATAGCCTCCTCTTCCA
XM_011654801 (*FeSOD*)	GAACTGAAGCCTCCTCCATA	CTCTATCTGTCTGTTAAGGTTGTC
D88649 (*cytAPX*)	GCAAGGATTCCAAGACTG	GTAACCTCAACAGCAACAA
XM_004146902 (*chlGR*)	TAGTGCTGTTCCTTGTGCTGTT	GCCGAAAGTTTGCTGTGTAGATG
AJ715498 (-*TUB*)	GCACTGGTCTTCAAGGAT	GTAAGGCTCAACGACAGA
AY372537.1 (*UBI-ep*)	ACCTTGTGCTCCGTCTCAG	CCTTCTTGTGCTTGTGCTTGAT
